# The structural shift and collaboration capacity in GenBank Networks: A longitudinal study

**DOI:** 10.1162/qss_a_00181

**Published:** 2022-04-12

**Authors:** Jian Qin, Jeff Hemsley, Sarah E. Bratt

**Affiliations:** School of Information Studies, Syracuse University, Syracuse, NY

**Keywords:** collaboration capacity, collaboration networks, GenBank metadata analysis, impact assessment, longitudinal study of collaboration networks

## Abstract

Metadata in scientific data repositories such as GenBank contain links between data submissions and related publications. As a new data source for studying collaboration networks, metadata in data repositories compensate for the limitations of publication-based research on collaboration networks. This paper reports the findings from a GenBank metadata analytics project. We used network science methods to uncover the structures and dynamics of GenBank collaboration networks from 1992–2018. The longitudinality and large scale of this data collection allowed us to unravel the evolution history of collaboration networks and identify the trend of flattening network structures over time and optimal assortative mixing range for enhancing collaboration capacity. By incorporating metadata from the data production stage with the publication stage, we uncovered new characteristics of collaboration networks as well as developed new metrics for assessing the effectiveness of enablers of collaboration—scientific and technical human capital, cyberinfrastructure, and science policy.

## 1. INTRODUCTION

Data repositories, software tools, and high-performance computing constitute key components of cyberinfrastructure (CI), which is established to facilitate and support data-intensive science. Data repositories store and manage scientific data and provide data submission, curation, and discovery services for sharing and reusing scientific data. Since the 1980s, the U.S. federal government has invested significant resources into building cyberinfrastructure, including data repositories and research data services. In parallel with the advancement of CI and growth of data repositories is a paradigm shift in science from empiricism, theory, and simulation to data (i.e., the fourth paradigm), as envisioned by Jim Gray (Gray, 2007; Gray, Liu et al., 2005) and subsequently articulated by Szalay and Blakeley (2009). Science today, small or large scale, is increasingly carried out through the distributed global collaborations enabled by CI.

The rapid increase in science data is attributable in no small part to the support provided by CI-enabled tools and services. The large number of tools for using the vast biomedical data available on the National Center for Biotechnology Information (NCBI)’s website underlines the importance of CI-enabled tools and services in data-driven science. GenBank is one of NCBI’s key data repositories and stores “massive amounts of genetic sequence data generated from evolving high-throughput sequencing technologies,” serving “more than 30 terabytes of biomedical data to more than 3.3 million users every day” (NLM, 2015). What is unclear in this grand picture of data-driven science is how this changing climate of science research has affected scientific capacity and the aggregation of the knowledge, skills, abilities, and technical facilities of individual scientists (referred to here as Scientific and Technical (S&T) Human Capital), as well as their networks of collaborative relationships (Bozeman, Dietz, & Gaughan, 2001). More broadly, there is also an unanswered question of how CI-enabled data services have impacted the increment of scientific capacity at individual, project, and institutional levels, and if there is any impact, how much it has affected the extent and rate at which scientists turn data into knowledge. Understanding these questions will require data beyond publication metadata to enable novel insights into the grand picture of data-driven science and CI-enabled research.

This paper reports the findings from a longitudinal study that uses the metadata from GenBank (Sayers, Cavanaugh et al., 2019) as the data source. We will first review previous research related to scientific collaboration networks and address the limitations of publication-based data sources in past research. As metadata from a data repository is a novel data source for studying collaboration networks, this paper attempts to provide the background of GenBank and its metadata and articulate on the suitability, feasibility, and possible issues in using this new data source to study data-intensive collaboration networks. Following the methods of data collection and processing, the analyses focus on the network structures and dynamics as well as their implications for the assessment of knowledge production and diffusion.

## 2. RELATED RESEARCH

Past research on scientific collaboration networks has generated a large body of literature that is scattered across scientometrics/bibliometrics, social studies of science, mathematics, physics (complex networks), information science, and science policy. Empirical collaboration network research has used almost exclusively publication metadata with varying sources and sizes, and with limited longitudinal time coverage. Theoretical research has also explored the statistical and mathematical mechanics of complex networks (Albert & Barabási, 2002; Costa, Rodrigues et al., 2007). Complex network theory has found wide applications in natural and social phenomena, including scientific collaboration networks (Barabási, 2009; Butts, 2009). This literature review section will focus on the complex collaboration networks research and rationalize the need for data-intensive study of collaboration networks and its implications to science policy research and research data practices.

### 2.1. Complex Collaboration Networks

Collaboration in research is typically measured by coauthorship in publications. Researchers in a collaboration network are called *nodes* or *vertices* and the relationships (i.e., coauthorship) between nodes are *edges*. Collaboration networks with very large numbers of nodes and edges together with variant weights of edges and other factors are highly complex, as nodes have uneven numbers of edges and the edges may vary in length between nodes. Such networks consist of clusters or communities of researchers, which are self-organized, may be interconnected in some ways, and evolve over time. Over the last 50 years, since de Solla Price’s work *Little Science, Big Science* (1963), scientific collaboration networks have been studied extensively from a wide range of disciplines. Newman (2001) collected and analyzed publication author data from MEDLINE, e-Print Archive, and NCSTRL, which represented the biomedical, physics, and computer science fields respectively. He found that these collaboration networks formed small worlds and the randomly selected nodes were typically separated by a short path of intermediate acquaintances. Scientific collaboration networks are essentially a kind of social network in which communities form through tightly knit groups (Girvan & Newman, 2002). Such a social aspect can be reflected in whom a researcher chooses to collaborate with and how such collaborations may enhance their S&T human capital (Bozeman & Corley, 2004). Barabási, Jeong et al. (2002) give an excellent summary of the research on collaboration networks, which include: Most networks have the “small world” property; real networks have an inherent tendency to cluster, more so than comparable random networks; and the distribution of the number of edges for nodes (degree distribution) “contains important information about the nature of the network, for many large networks following a scale-free power-law distribution” (p. 591).

The CI-enabled research environment led to a shift to what has been called the fourth paradigm of science, an era that is characterized by distributed global collaboration, data-intensiveness, and reliance on high-performance computing (Szalay & Blakeley, 2009). Large data repositories have been built in the last three decades for researchers to submit, manage, share, and reuse data. For many scientific disciplines, submitting to a repository has become part of the regular research process and been made as policy mandates (NIH, 2021; NSF, 2020). As the science paradigm shifts and data management and sharing policy mandates blurred the boundaries between data professionals and researchers, researchers have been devoting more time to data processing and analyses. The cause of this blurred division of labor stems from the work needed to make raw data clean. That is, data usually cannot be directly fed into algorithms without preprocessing, transformation, and sometimes meshing with other data sources (Kamath, 2009). The impact of such paradigm shift on collaboration networks is largely unknown and publication coauthorship alone would be insufficient to address. The CI-enabled links between publications and data sets have created a ripe condition for studying complex collaboration networks on a large scale by integrating metadata from data submissions.

### 2.2. Theories and Models

The study of complex networks has traditionally used graph theory, but in the last 50 years statistical methods have gained increasing significance in this research field. Questions of interest for complex network researchers include the typologies and properties of complex networks, interaction between these two components in a network, and the tools and measurements for capturing “in quantitative terms” the underlying organizing principles of real networks (Albert & Barabási, 2002). Well-known theories include those of random graph, per-colation, small-world networks, scale-free networks, networks with community structure, and evolving networks, for which Albert and Barabási (2002) and Costa et al. (2007) provided exhaustive surveys.

Three of the theories/models among those reviewed by Albert and Barabási (2002) and Costa et al. (2007) are the Watts-Strogatz model of small-world networks (Watts & Strogatz, 1998), the Barabási-Albert model for scale-free networks (Barabási & Albert, 1999), and the theory of evolving networks (Albert & Barabási, 2002). In the discussion of each of these theories and models, Albert and Barabási (2002) used the average path length, clustering coefficient, and degree distribution, among others, to explain the statistical mechanics of these theories and models, which are considered as three robust measures of a network’s topology. Network theories and models have been applied in studying collaboration networks in biology, ecology, and physics, as mentioned in the previous section. Several properties of scientific collaboration networks have been identified in these studies: Small worlds are common in scientific communities; the networks are highly clustered; and biomedical research appears to have a much lower degree of clustering compared to other disciplines such as physics (Newman, 2001). The evolution of scientific collaboration networks shows that the degree of distribution follows a power law and key network properties (diameter, clustering coefficient, and average degree of the nodes) are time-dependent; that is, the average separation decreases in time and clustering coefficient decays with time (Barabási et al., 2002).

### 2.3. The Data Gap

Studies of scientific collaboration are abundant in scientometrics and information science scholarly journals. Many of them are often limited in that the data used are filtered by discipline and period from a single database and almost exclusively use publication-based authorship data, as seen in the studies cited above. The limitations of data source and variant timescales make it very difficult, if not impossible, to generate data sets that can be meaningfully reused or integrated with other data sources for understanding the complexity of scientific collaboration networks. Metadata in scientific data repositories offer a new breed of data source for studying research networks. Their large scale and continuous time coverage provide a rich testbed not only for developing models and theories but also for meshing other related data sources to examine and interpret complex collaboration networks from more dimensions.

## 3. THE BACKGROUND OF GENBANK

GenBank was conceived in 1979 by a group of biologists and computer scientists at a meeting held at the Rockefeller University in New York. The meeting participants agreed on “the necessity to create a national, computerized database” (Strasser, 2008, p. 537). Three years later the Los Alamos Sequence Library became the cutting-edge repository—GenBank—for curating nucleic acid sequence data (Cinkoski, Fickett et al., 1991). Soon after, the sequence data started to grow exponentially as the computer technology and network availability rapidly advanced in the second half of the 1980s. Meanwhile, nucleotide sequencing methods and technologies have evolved from the first generation represented by “Sanger sequencing” to Next Generation Sequencing (NGS), which allowed many parallel sequencing reactions at a much lower cost, namely high-throughput sequencing (Heather & Chain, 2016). During this period, the sequence data processed by GenBank grew from 1.38 million nucleotides in 1984 to 14.1 million in 1990 (Cinkoski et al., 1991).

Early data entry into GenBank relied on curation staff who performed extraction of nucleotide sequences from published articles and made them available in electronic form to researchers. The rapid increase in the volume of nucleotide sequence data soon made it clear that this model could not keep up with the growth of sequence data, as it was labor intensive, and the publishing of these data lagged far behind their generation. In addressing this problem, GenBank worked with journal editors to develop policies to make direct submission of sequence data to GenBank a requirement for publishing a paper. This policy mandate, together with automated data processing, not only reversed the data flow, which was originally from journal articles to GenBank (Cinkoski et al., 1991), but also pioneered the incentive mechanisms for data sharing. Another significant driving force for GenBank’s data growth is the Human Genome Project (HGP, https://www.genome.gov/human-genome-project), which started in 1990 and was completed in 2003. Six years into the HGP, countries participating in this international effort reached consensus on the timely release of sequence data through the Bermuda Principles, which established policies on sequence data quality standards, sequence submission and annotation, and sequence claims and etiquette to ensure the prepublication sharing and rapid disclosure of sequence data (BERIS, 2019; Cook-Deegan & McGuire, 2017; Maxson Jones, Ankeny, & Cook-Deegan, 2018). If the development of NGS technology accelerated the increment of the volume and kinds of sequence data and shifted data generation toward more analyses (Alekseyev, Fazeli et al., 2018), then the journals’ requirement for data submission before manuscript submission and the Bermuda Principles cultivated the data sharing culture, an impact that goes far beyond GenBank.

**Figure 1.**
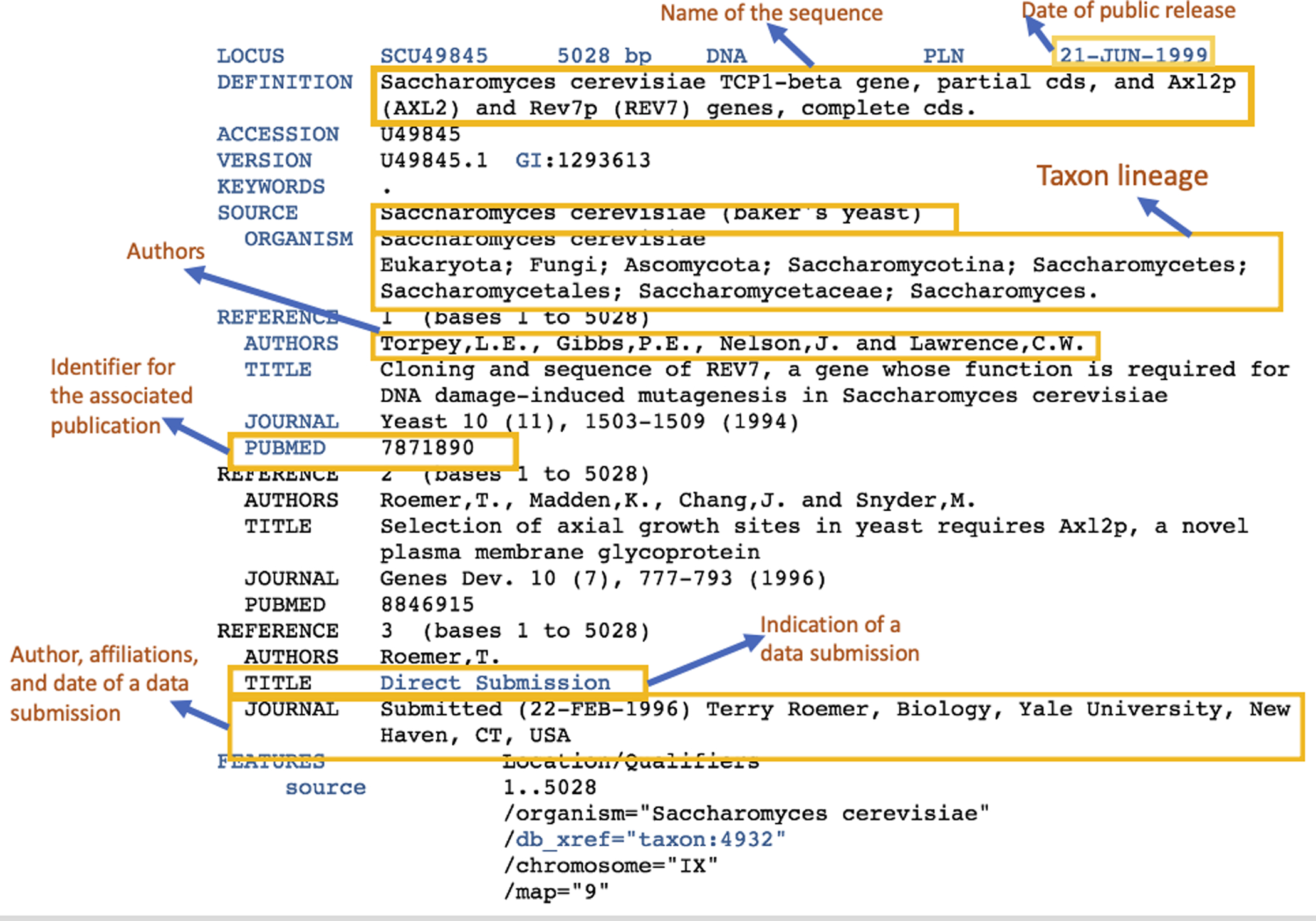
The metadata section in a GenBank annotation record.

The GenBank records are acquired in two ways: direct submission by individual researchers using tools such as BankIt (https://www.ncbi.nlm.nih.gov/WebSub/) and Submission Portal (https://submit.ncbi.nlm.nih.gov/), and batch deposit from sequencing centers by sequence types (Benson, Karsch-Mizrachi et al., 2011). The author field in these tools is designed to support multiple author entries in an annotation record. The public display of metadata section in GenBank annotation (Figure 1) does not show all the data authors, but they are in the released files on the FTP server.

Although the advances in sequencing technology liberated researchers from performing sequencing work themselves, the researchers themselves continued to act as authors of the data submissions. In one of the data sets we created by matching the NIH funding records with the GenBank records related to infectious diseases, we randomly selected 55 GenBank records. We used this sample to examine the authors who submitted the sequences to GenBank and how they were related to the principal investigators (PI). The funding data set was extracted from the NIH RePORT database, which contains information on PIs, publications, and affiliations. We mapped the funding records to GenBank records by PubMed article ID (PMID), which allowed us to track submission author’s affiliations and roles by triangulating with multiple sources of information, including affiliation and acknowledgment in the article, institutional and personal websites, LinkedIn, and researcher’s curriculum vitae/resumé. The records examined represent only a small fraction of the GenBank records; hence we do not have the generalization power of the whole data collection. Nevertheless, they offer some insights into who the submission authors are and what roles they may have played. Table 1 presents the summary of the findings from the manual checking of 55 records at three different time intervals.

We observed that many submission authors in this sample were also publication authors, while the PI was listed in publications more than half of the time. Through triangulation among the multiple sources mentioned above, we found that when the submission authors and the PIs appeared in both submissions and publications, they were more likely than not in a PhD advisee–advisor or postdoc–mentor relationship. In this context, the first author in data submission and publication was usually the doctoral student or postdoc. When the PIs were not included in the publication or submission, it seemed that they often held a position such as a director of a large laboratory or a government research staff position that did not allow them to engage in the project enough to be given the credit. In some cases, the submission authors were visiting scientists with their own grant and project but needed to use the research facility of a given PI. Although we observed in several acknowledgments that the sequencing was performed outside of the submission authors’ labs, this did not change the fact that submission authors were mostly researchers themselves who were also actively engaged in publication activities.

Sequence data submitted to GenBank will be assigned an accession number and reviewed by GenBank staff for quality assurance purpose. A GenBank annotation record contains metadata for identifying and describing the creators and characteristics of the sequence data, including authors who are included in the direct submission field, date of submission, data of public release, and publications associated with the sequence submission, as well as the molecular attributes of the sequences, such as locus, taxon lineage, and features (Figure 1). It is worth pointing out that the time between the date of submission and date of public release provides an important piece of information about the data-to-knowledge production. A GenBank record has two sets of authors: those in publications (references) and those in direct submissions of molecular sequences (i.e., the data authors). An author may or may not appear in both spaces, though it is likely that many authors reside in both the publication and sequence submission metadata. Because the act of data submission represents a stage in a research life cycle earlier than publication, examining the metadata about sequence data submissions and subsequent publications provides an opportunity to uncover how collaboration networks evolved “in action” and gain insights into research collaboration that publication authorships alone would have been unable to offer.

One caveat in using metadata from GenBank to study collaboration networks is that the publications associated with data submissions are not representative of the full publication productivity of researchers because GenBank is not a publication repository. Therefore, metadata for data submissions are more suitable for studying relationships between publication and data submission networks than publication productivity. The data about sequence submissions, for example, the dates of sequence submission and public release, as well as related dates of patent applications and publications, allow for the creation and testing of new metrics for evaluating the impact of cyberinfrastructure, science policy, and S&T human capital on the biomedical research enterprise.

## 4. METHODS

### 4.1. Data

GenBank data is hosted on an FTP server at NCBI. The GenBank flat file release 229 (cutoff date December 15, 2018) consists of 3291 files in compressed format, each of which ranges between single digit to three-digit megabytes. We downloaded all the annotation records from 1982 to 2018 and extracted the metadata section in January 2019. The extracted metadata were then parsed into a relational database (we excluded the genetic sequence data, which comprised about 80% of the data volume). The data download and processing workflow included the following steps:

- Download one compressed sequence file from the FTP server.
- Decompress the file.
- Extract the metadata section from each record in the file.
- Save the metadata records to a buffer space.
- Delete the downloaded file.
- Parse the metadata into a database.
- Repeat the workflow for the next compressed file on the FTP server.

A computer program was created to automatically complete these steps in a batch style. We set up a data server with the necessary software and storage space for the GenBank metadata extractions. This process resulted in 227,905,057 annotation records minus the sequence data, in which 44,480,172 publications were referenced. This data collection also includes 42,511,832 patent references.

Author names in this GenBank metadata collection were disambiguated by using the Kaggle solution from Chin, Zhuang et al. (2014) and by cross-checking the results with author metadata from Web of Science, SCOPUS, and Microsoft Academic Graph. After the disambiguation process, the data collection resulted in 877,134 unique author names (nodes), of which 519,719 are in the publication network, 523,013 in the submission network, and 214,197 are unique scientists in the patent network.

We grouped the data by year and then, for each year, we constructed two networks: a publication coauthor network and data submission coauthor network. For each network, we built a data set that included information such as the year, if it was a publication or data submission network, how many publications (data submission) there were, and the number of authors, as well as network statistics such as degree centrality and clustering coefficient. We also looked at the distribution of degree centrality for each network. The degree centrality of all these networks, except the first few years, follows a power law. Research has shown that the shape of a power law distribution can be a useful signal that reflects information about the network (Hemsley, 2016). As such, we use the power-law shape parameter in iGraph (Csardi & Nepusz, 2006), which is an R package devoted to social network analysis, and stored that in our data as well. This data collection went through parsing, name disambiguation, slicing by year, and edge list generation and was used to compute the statistical properties for screening and analysis. For additional analysis, the publication and submission networks for each year were merged and the calculations rerun.

### 4.2. Measure for Collaboration Capacity

The inclusion of data submission metadata created an opportunity for examining a new aspect of collaboration networks: Collaboration Capacity (CC). In the context of this paper, we define CC as the ability of an individual, group, or institution to assemble and effectively use the S&T human capital in collaborative research. We assume that the greater the S&T human capital a researcher can accumulate or assemble, the more opportunities and resources they can garner to collaborate with other researchers and the more likely the S&T human capital will be used more effectively. This means that CC measures not only how much S&T human capital a person may accrue but more importantly, how effectively they can utilize the S&T human capital as well as the support provided by cyberinfrastructure and science policy to increase productivity and innovations. Because collaborative research starts well ahead of a coauthored publication, the trace data that document collaboration prior to publication, namely, the data submission records in science data repositories, can provide insights into the assessment of research performance and impact.

One of the measures we tested for CC is the number of new collaborators an author added to their coauthor list in a period. To compute the value of CC for individual authors, a sample of authors was selected by following two criteria: Authors eligible to be selected should be located in the elbow section of the L-shaped distribution (which is the pattern for all years; see Figure S1 in Supplementary Materials); that is, not those with extremely high number of publications or in the long tail, which was determined as between 1–50 publications; and an author must have published at least once in a 3-year window starting from 1997, namely, 1997–1999, 2000–2002, 2003–2005, etc., to be selected. A random selection of authors with these two criteria generated a sample of 6,503 authors in 10 3-year windows between 1997 and 2017. The computation of CC was performed on all 6,503 authors. The following steps were taken to calculate the value of CC:

1. Find all coauthors of an author who had collaborated each year during 1997–2017. If an author was inactive in a given year, they would not have any coauthors that year.
2. Collapse this timeframe into windows of 3 years each. Now each window has a list of all authors with whom an author collaborated in that 3-year window.
3. Remove any duplicate authors that may have appeared in the list. For example, if an author collaborated with an author twice in one window, they will be counted just once.
4. CC values were calculated in two ways:
  - Noncumulative CC: this value is obtained by counting how many *new* authors an author added as compared to previous window. For example, if an author collaborated with three authors A, B, and C in window 1 and three authors A, D, and F in window 2, this author would have two new authors (D, F) in window 2. Therefore, the CC value for window 2 is 2. The resulting CC value is the average of all windows, hence noncumulative collaboration capacity for that author.
  - Cumulative CC: this value measures how many new coauthors an author added in a given window as compared to *all* previous windows. For example, suppose an author collaborated with two authors A and B in window 1, two authors B and C in window 2, and 2 authors A and D in window 3. The CC value for window 3 will be just one because the author added only one new author (D) in window 3 as they had already collaborated with author A in window 1. If it were noncumulative collaboration, the value for window 3 would be 2, as both A and D are new authors as compared to window 1. The average of all windows is used as the average cumulative collaboration capacity value for that author.

## 5. RESULTS

### 5.1. Collaboration Networks in Time

**Figure 2.**
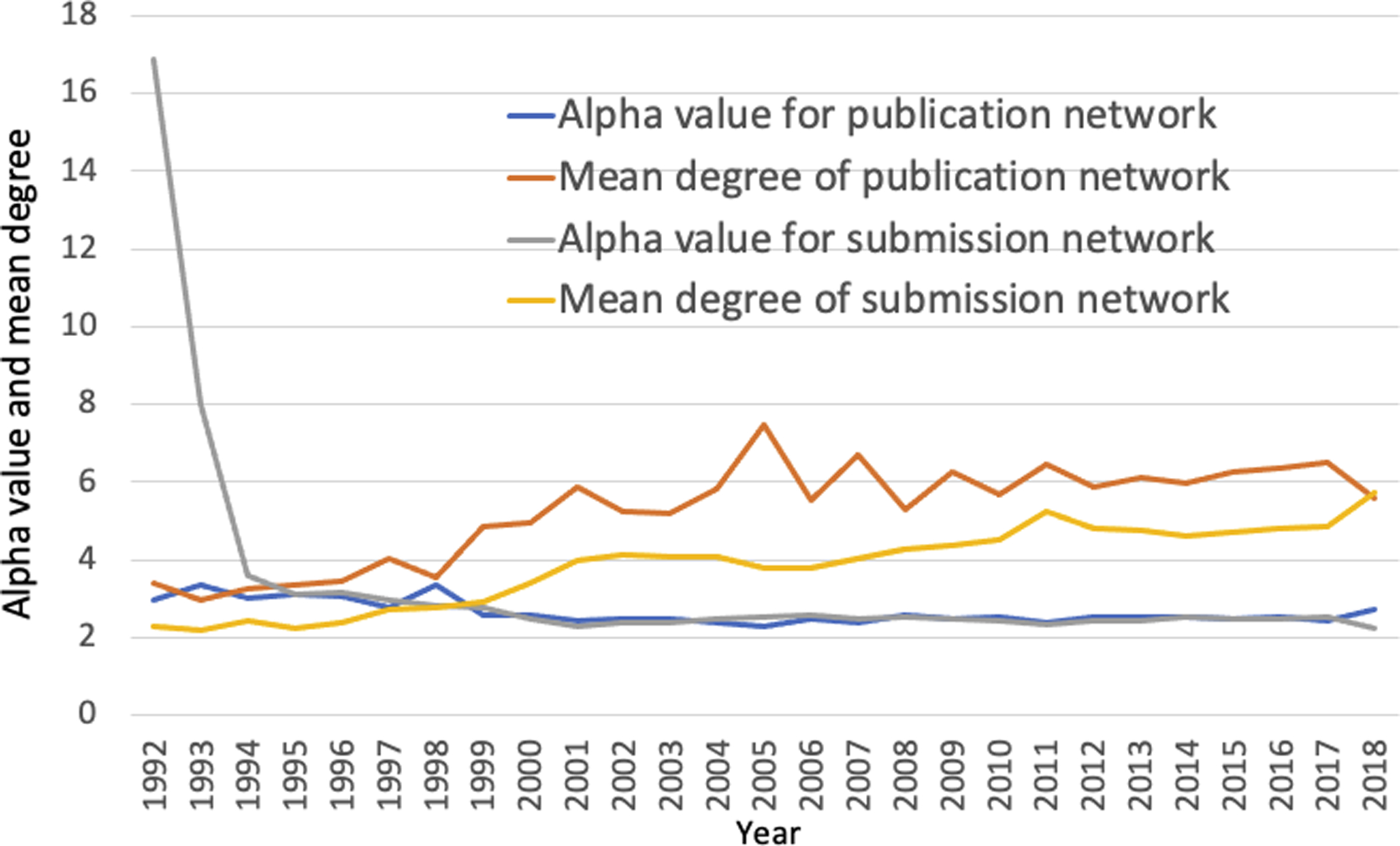
Distributions of alpha values and mean degrees for both publication and sequence data submission networks in GenBank 1992–2018. The alpha values for both networks appear to be almost identical, while the mean degree values for publication network have been consistently higher than that of the submission network. (The data used to generate this chart are in Table S1. In this paper, table and figure numbers with an S mean they are in Supplementary Materials).

GenBank started operation in 1984. It took about 8 years for the growth in data submissions to take off. Data before 1992 were merged into 1992 due to the sporadic nature of direct submissions. Figure 2 shows that the mean degree (average number of connections an author has) for the GenBank publication network doubled from a mean of 3 to 6 by 2018. At the same time, the mean degree for sequence data submission networks almost tripled (Figure 2).

The publication network displayed a scale-free property from 1999 onward while the data submission network showed a scale-free property earlier in 1997 (Figure S1 and Table S1). A Kolmogorov-Smirnov test (see Table S1) confirms that the degree distribution of GenBank networks fits a power law distribution (Clauset, Shalizi, & Newman, 2009). A further examination of the data reveals that when we merge the GenBank publication and data submission networks, the result also has a power law distribution after 1998 (Table S2). Analysis of the combined publication and data submission networks displays a trend of increasing percentages of nodes belonging to the giant components throughout the whole 27-year span. A giant component is a set of nodes in a graph that are connected directly or indirectly and an indicator for the connectedness of nodes in a graph. The size of the giant component in GenBank (publication and submission networks together) grew from 43.7% in 1992 to 82.2% in 2012, the highest point in all years, before dropping off its peak by 15% by 2018 (Table S2), an indication that the networks became more interconnected over time.

A prominent property in scale-free networks is that they follow an 80/20 rule (Barabási, 2016). In the case of GenBank combined networks, the degree distribution of authors clearly presents this property. In Figure S1, the red colored points represent authors only in the data submission network, blue points represent authors only in the publication author network, and purple points represent authors who were in both publication and submission networks. The degree distribution in these plots appears highly skewed, following an L-shape. That is, a very small number of authors had very high degree centrality in the publication or submission networks or both, while the majority of authors tended to have a very low number of connections. As time went on, the number of authors only in the data submission networks (red) and in both networks (purple) grew, while the number of authors only in the publication network grew much more slowly.

However, three strata of degree distribution among the authors can also be seen in Figure S1: a majority of authors remained at the bottom level (<10 links), the middle group ranged roughly between 10 and 500 links, and a very small number of authors had over 500 connections. Also, the red tail on the plot suggests that those in the data network only tended to have the smallest number of links, while those nearer the top, or those with the most connections, tended to work in both networks. In fact, the plots shift from mainly blue (publication only) to mainly purple (both networks), with a red long tail, over time, implying that more activity and people were engaged in the data work. It also suggests that more actors who were publishing were also engaged in the data work.

### 5.2. Structural Shift

**Figure 3.**
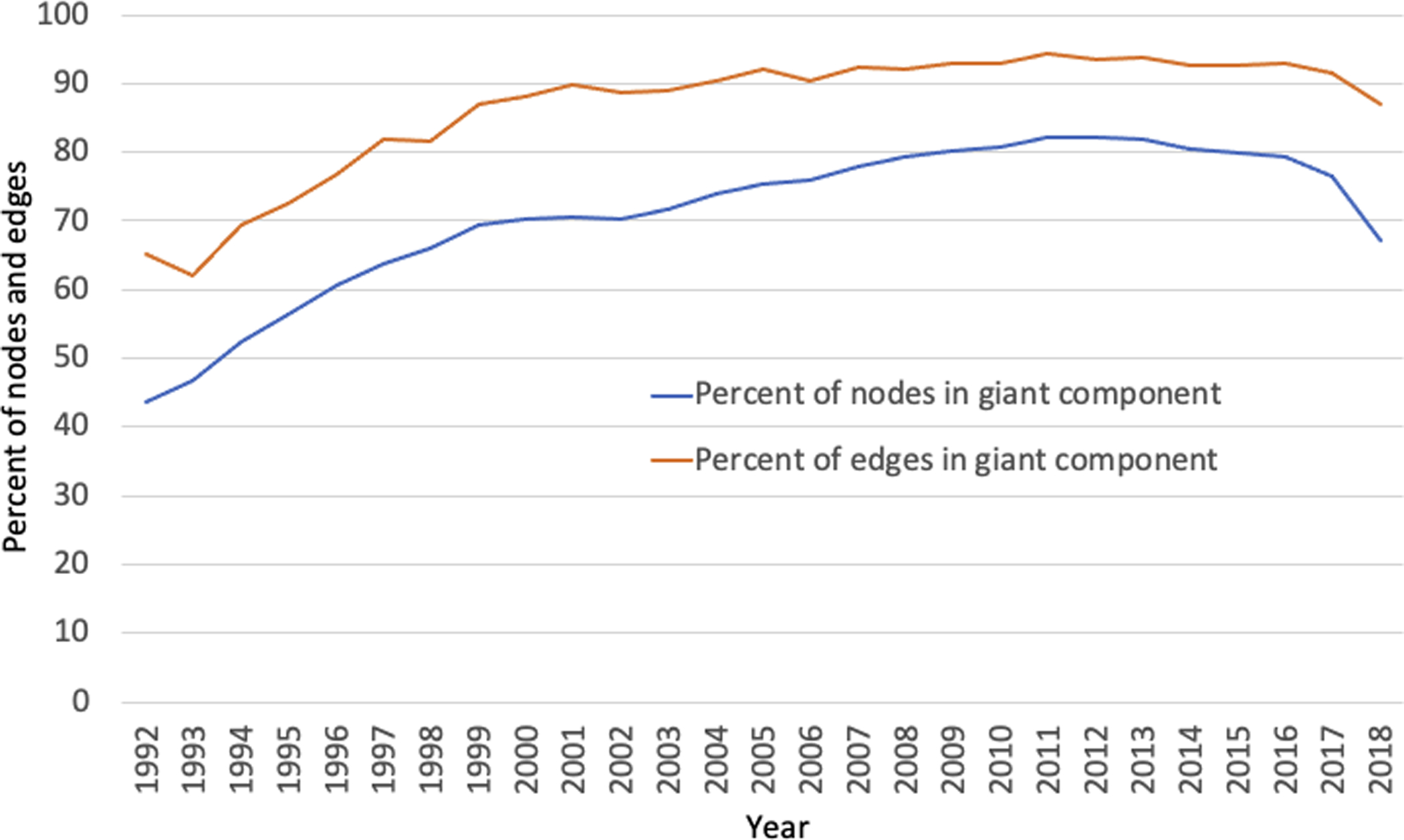
Giant component size changes from 1992–2018 have been steadily growing. The growth in the percentage of edges has outpaced that of the nodes. See Table S2 for the data used to draw this plot.

As noted above, and shown in Figure 3, we observed that the percentage of nodes in the networks that were in the giant component tended to increase. However, in 2018 it decreased to near 1998–1999 (67%) levels. The percentage of edges in the giant component remained high and had only a slight decline.

**Figure 4.**
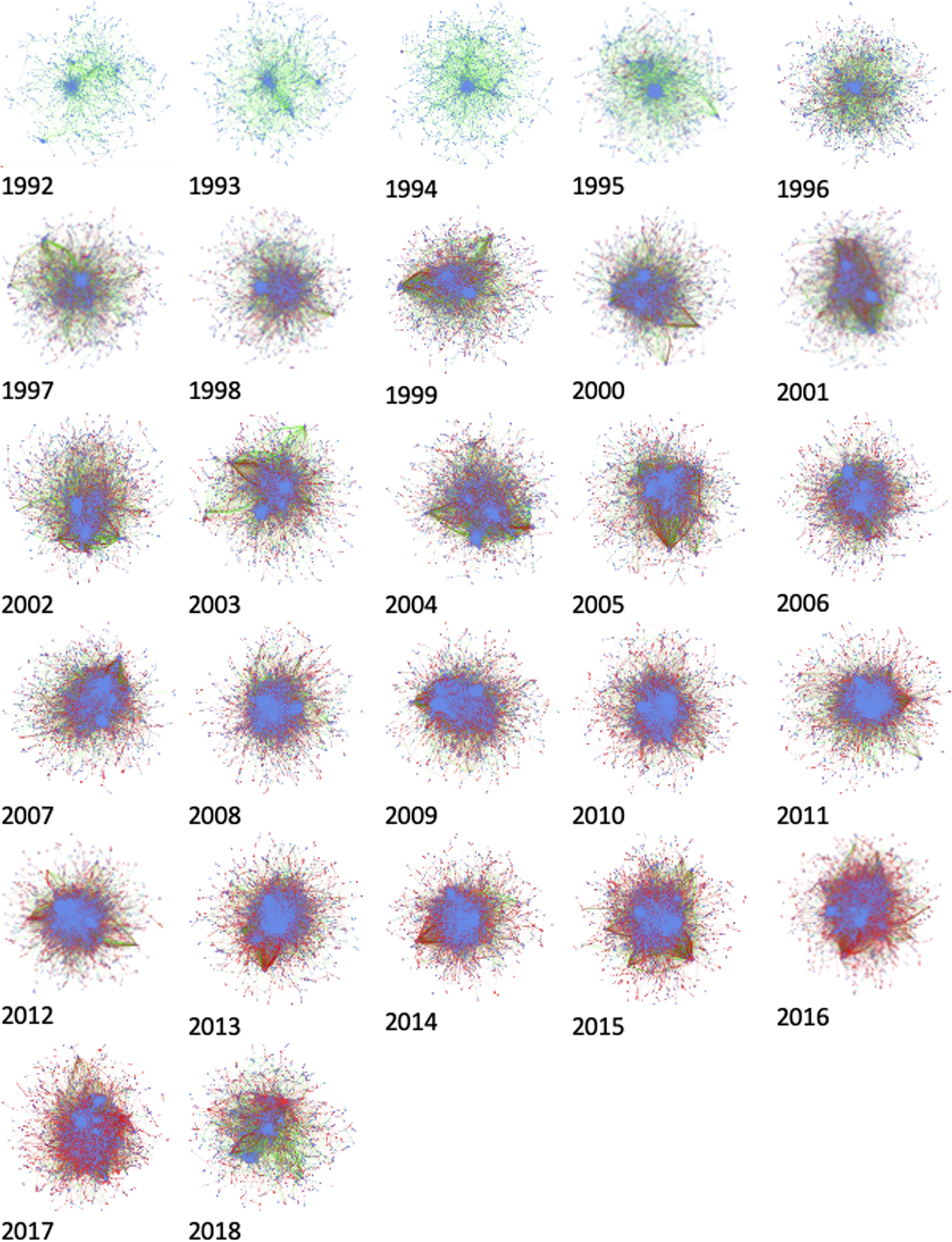
GenBank network visualization from 1992–2018: Each network represents 1 year of the data and includes the merged data submission and publication coauthor networks. Nodes that only showed up in the publication network are blue with green links. Nodes that only showed up in the data submission network are dark red, with red links. Nodes that showed up in both networks are purple with dark purple links between them. To observe the main structures, we are focused on the giant component for each year; thus isolates and disconnected clusters have been removed. Larger-size visualizations of yearly network structure changes can be seen from Movie S2 in Supplementary Materials.

Even though quantitatively the percentage of nodes in the giant component in 2018 dropped, the structure of the network in 2018 was quite different from that in 1998 and 1999. As observed in Figure 4, the network displayed a publication-centric (blue nodes) structure during 1992–1995, and after that initial period, the growth of data submission nodes (in red) was increasingly visible and even started to overshadow the publication author nodes in the last few years. Starting from 2008, the network appeared to be less concentrated on a few dominant hubs. More regional clusters or communities emerged with strong local connections (the red dots on the visualization represent the density of connections). It is notable that some highly connected node clusters emerged from outside the giant component and these clusters of nodes occurred mainly among the data submission nodes (Figure 4). A possible explanation is that, as the number of edges remained steady, a decrease in the number of nodes means more links between fewer people in the giant component and the nodes that shifted outside of the giant component to form new, more tightly connected local clusters could be the reason for the shrinking giant component.

This phenomenon seems to coincide with the decrease in clustering coefficient for both publication and data submission networks (Table S3), signaling a flattening network structure during the entire period. This trend accelerated at a faster pace starting around 2006. All these network behaviors implicate a structural shift in GenBank collaboration networks that went from densely clustering around a small number of hubs to dispersed local clusters with stronger ties inside the clusters. The fact that the node percentage in the giant component had a big dip in 2018 can be seen as an echo of the steady drop in grant-eligible young PIs in NIH R01 grant awards (Levitt & Levitt, 2017; Pickett, Corb et al., 2015).

**Figure 5.**
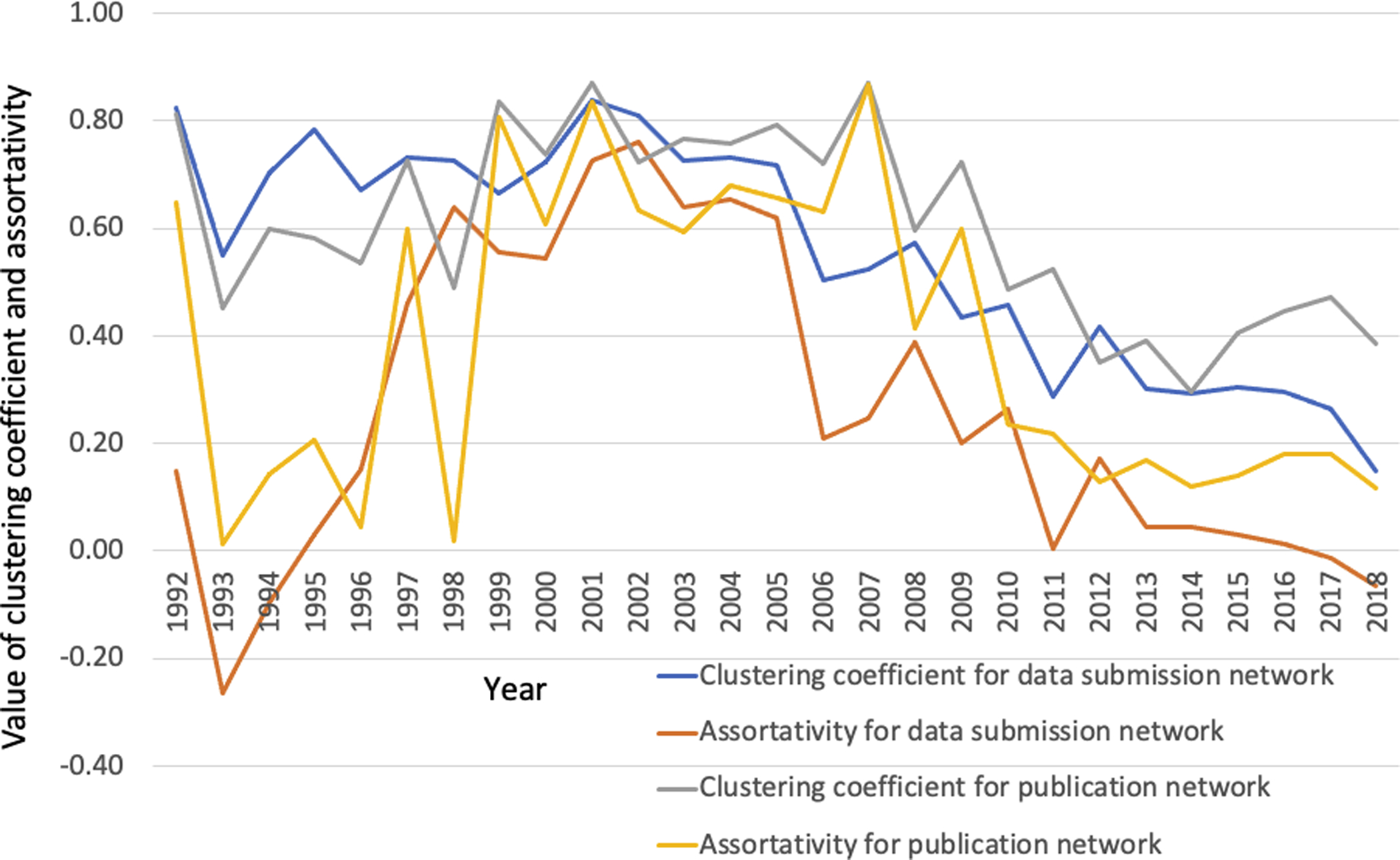
Distribution of clustering coefficient and average assortativity for publication and data submission networks from 1992–2018. (See Table S3 for data used to generate this plot.)

The observed structural shift is supported by two statistical properties of GenBank networks: the clustering and assortativity coefficients. As shown in Figure 5, the clustering coefficient for both publication and data submission networks followed a downward trend, starting around 2007. It may be considered as a sign that GenBank networks were no longer dominated by a small number of highly connected “hub” nodes, but rather, the networks tended to be flatter, with more scattered, smaller clusters interconnected through a few bridge nodes. A similar trend is also visible in assortativity for both publication and submission networks, though with more turbulent fluctuations. Assortativity in networks measures the likelihood that nodes with similar properties link to other nodes with those properties. The measure ranges from −1 to 1, with 1 = perfect assortative mixing, 0 = nonassortative, and −1 = completely disassortative mixing. The fact that the assortativity coefficient for data submission network was near or below zero from 1993–1995, then above, and below zero again from 2016–2018 is an indication that the data submission network went through a structural shift from disassortative to assortative mixing then back to disassortative mixing (Figure 5). In other words, author nodes were more likely to connect with those having similar properties, then dissimilar and then similar again over time. At the same time, the global network was flattening structurally (i.e., there were more locally tightly linked clusters that had connections to “hub” nodes through bridging nodes), which we see as more evidence for structural shift.

**Figure 6.**
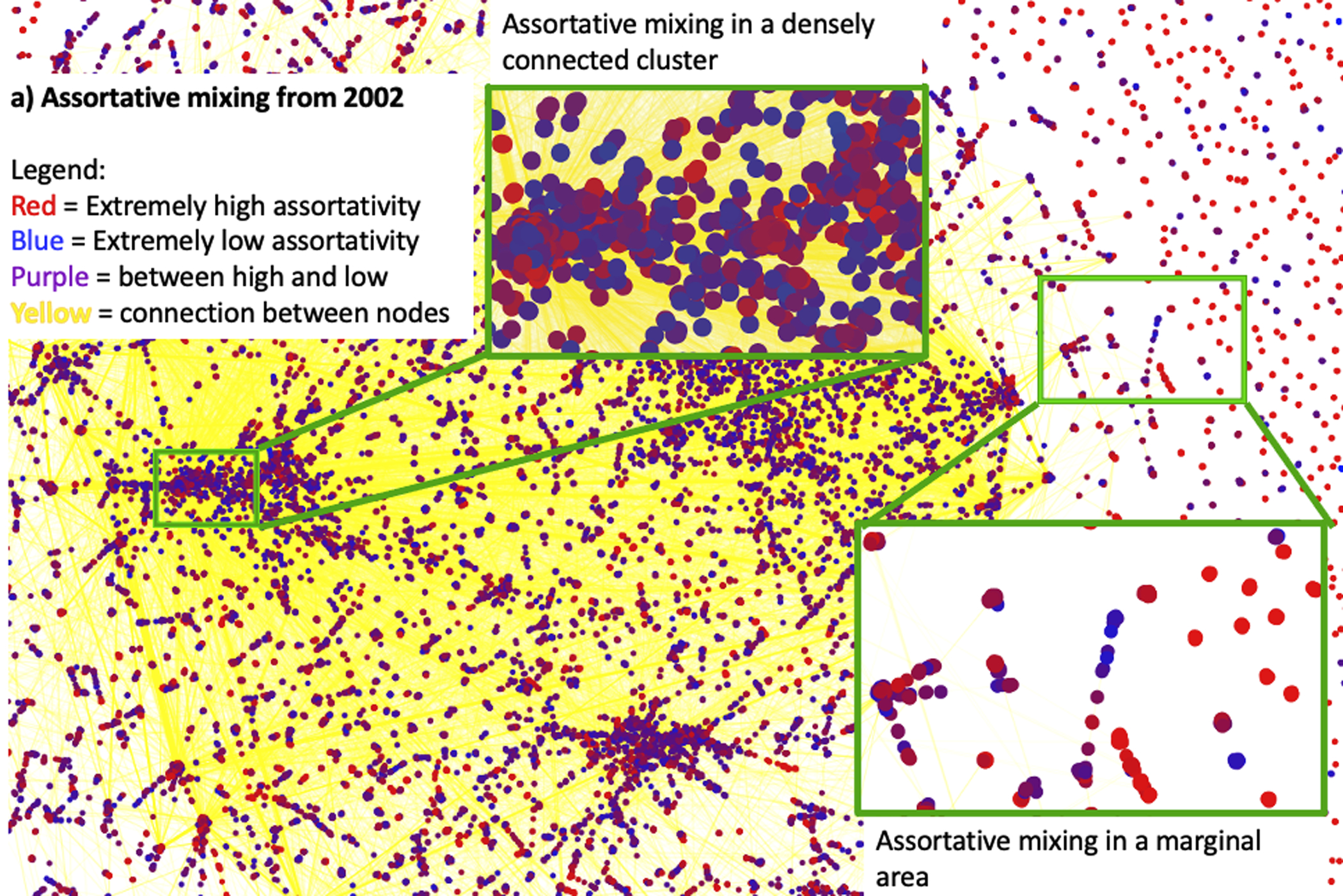

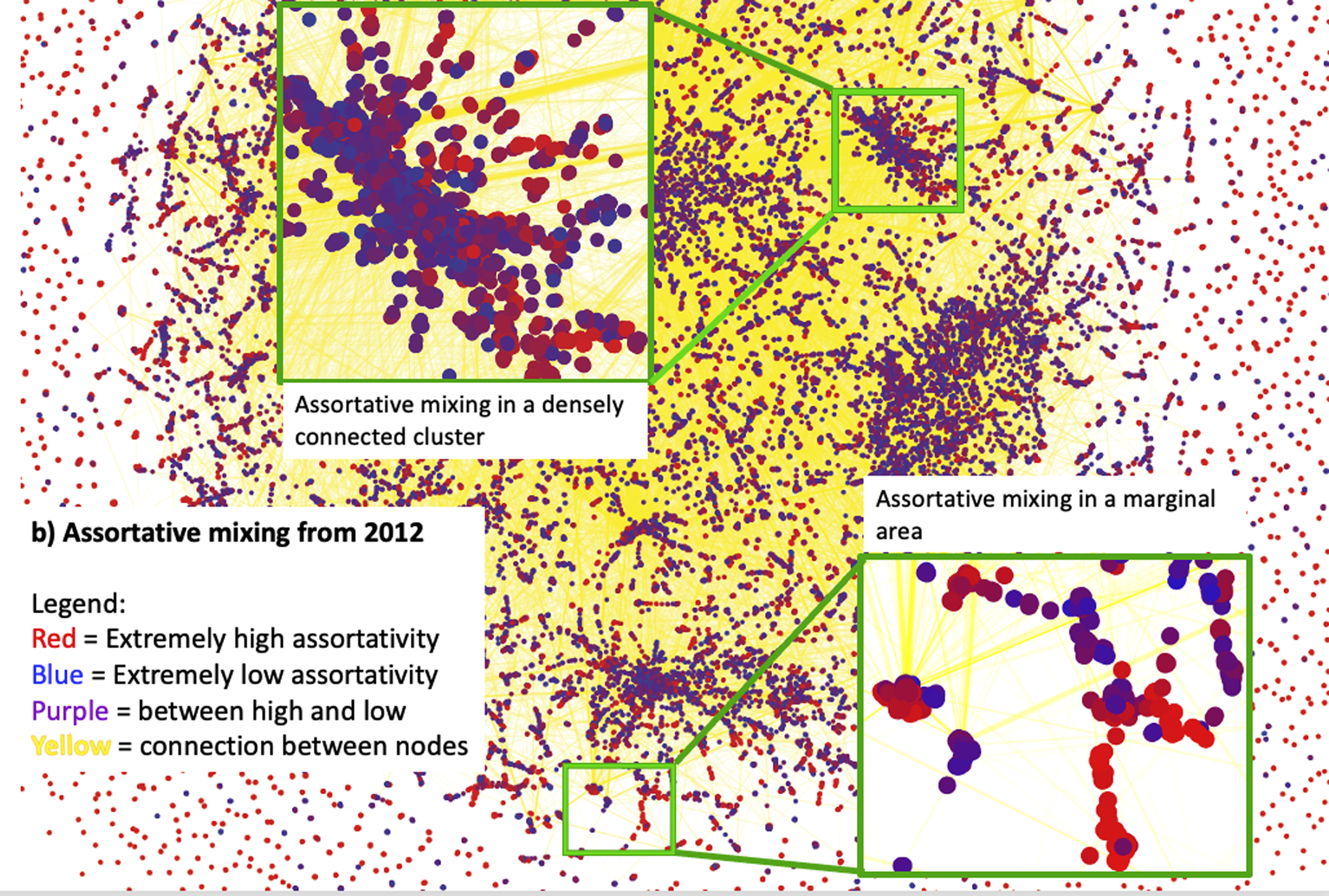
Assortative mixing for 2002 and 2012: (a) A densely connected cluster and a sparsely connected region in 2002. There appear to be few connections between the nodes with high assortativity mixing (in red) and those with low assortativity mixing (in blue), similar to the outer region with sparsely connected nodes. (b) The densely connected cluster shows stronger mixing between high and low assortativity in 2012, while the sparsely connected outer region appears to have little mixing between high and low assortativity.

To explore further the detail of GenBank network assortative mixing, we selected the year 2002, which had the highest assortativity coefficient value among all years, and 2012, a decade on from 2002, to see how assortative mixing, and thus the network structure, changed. We computed the assortativity values by using the multiscale mixing algorithm (Peel, Delvenne, & Lambiotte, 2018). The network graphs in Figure 6 show author nodes in both publication and data submission networks (the combined network). Colors indicate the level of assortativity, with red being the highest and blue the lowest in assortativity mixing. The assortativity coefficient peaked in 2002, then dropped steadily afterwards. Table S3 shows that, on average, the data submission network coefficient was 0.761 and the publication network coefficient was 0.634 in 2002, while these numbers dropped to 0.172 and 0.128 respectively in 2012.

The detailed regions in Figure 6(a) show that extremely high and low assortative mixing coexisted among densely connected nodes, while in outer regions nodes were sparser and had fewer connections (mainly red periphery nodes). There was a tendency for nodes to connect to more similar nodes. Similar assortative mixing remained a decade later in 2012 (Figure 6(b)); however, the center of clusters tended to concentrate with high assortativity nodes, while the nodes in the outer regions were more homogeneous, with much less assortative mixing in 2012.

### 5.3. Collaboration Capacity

**Figure 7.**
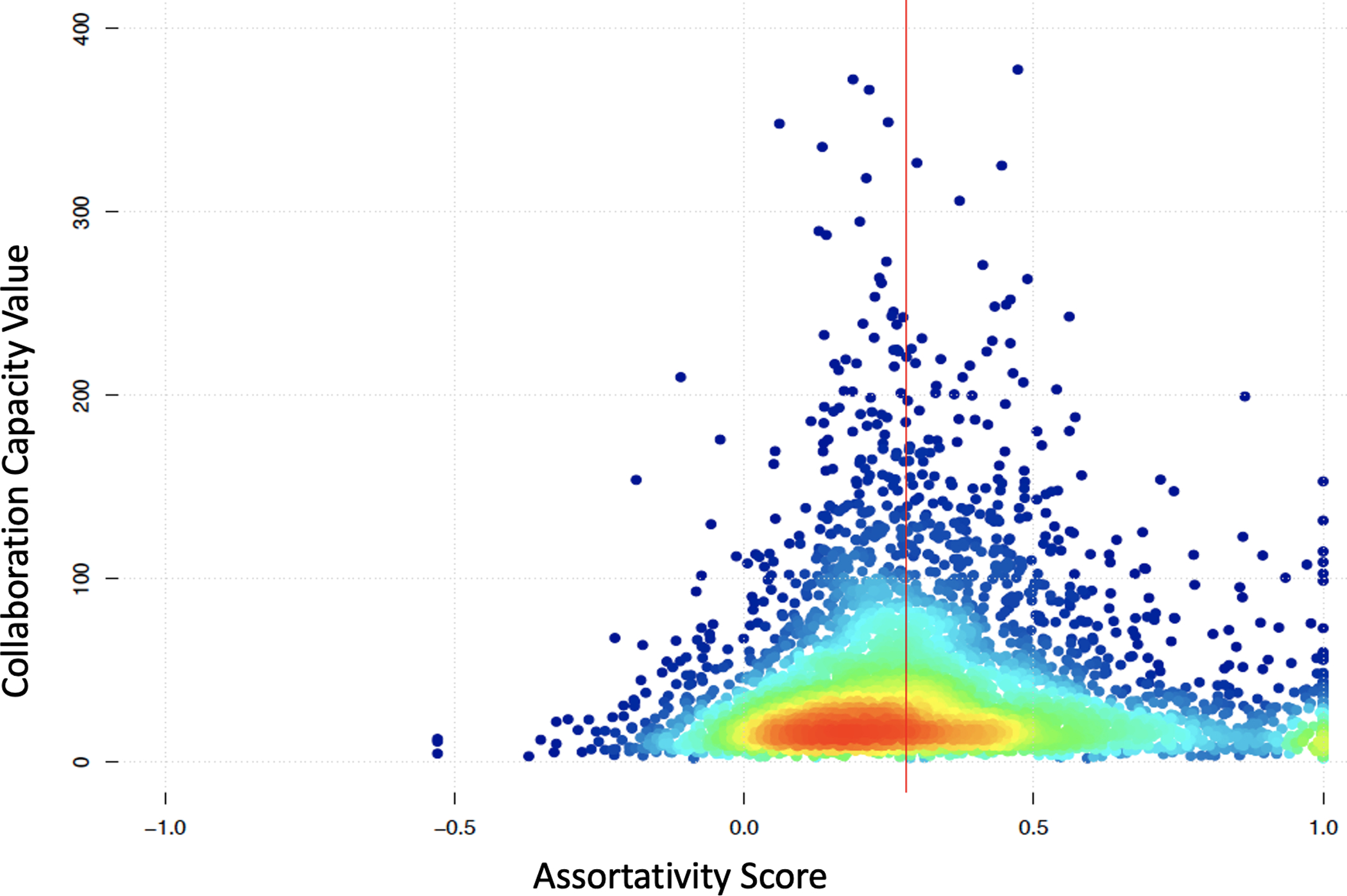

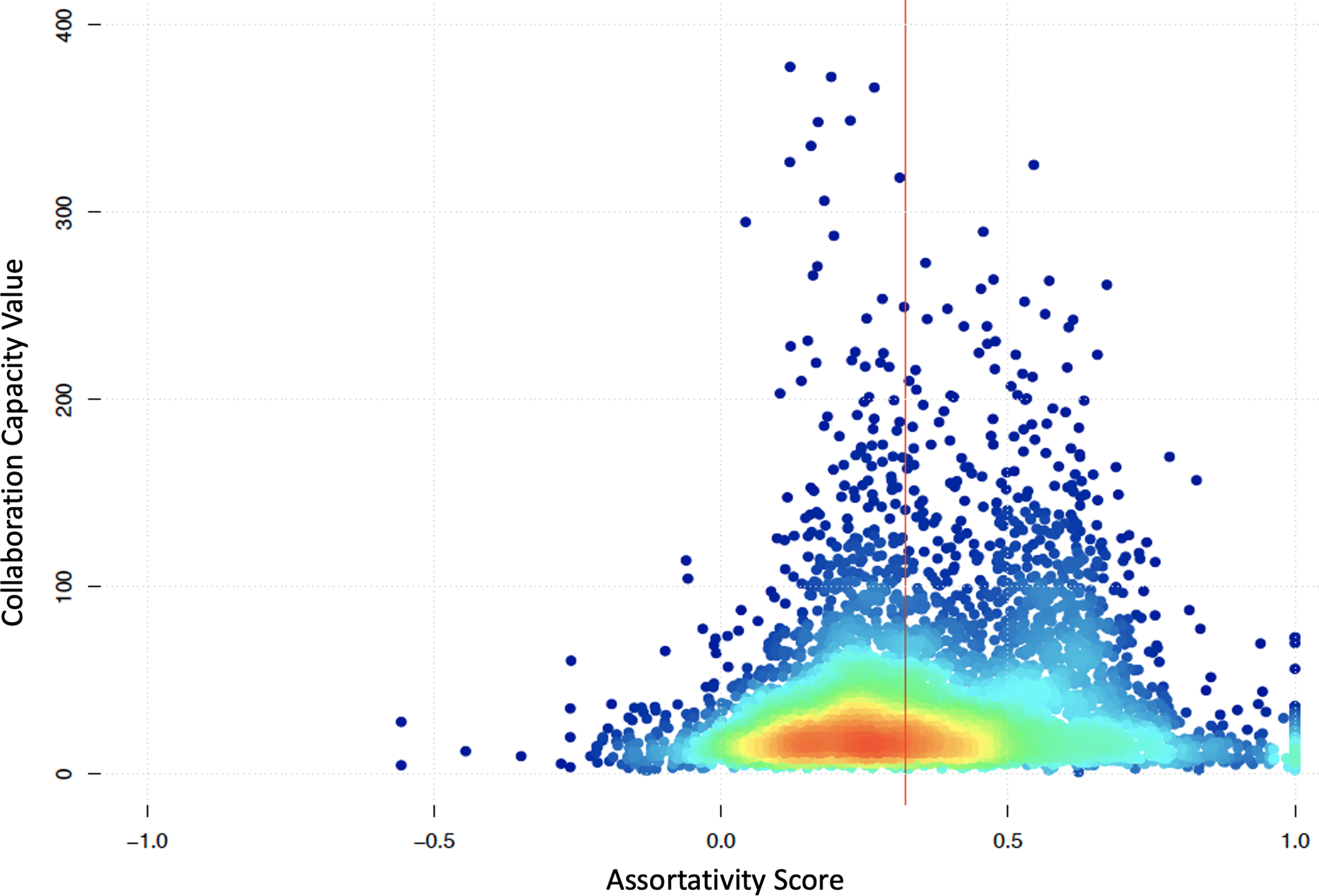
Assortativity vs. collaboration capacity. The relationship between assortativity and collaboration capacity is consistently positive, as reflected in the 2002 and 2012 snapshots of the author-level statistics. The heat map color spectrum shown in the graphs shows the density of the values, that is, the frequency of the values, around the mean (vertical red line of ~0.3 assortativity score).

Using the number of new collaborators acquired over a period as one of the measures for collaboration capacity (Qin, Hemsley, & Bratt, 2018), we drew a random sample of 6,503 nodes from the networks between 1997 and 2017 and plotted their assortativity coefficient scores against collaboration capacity as measured by the number of new collaborators acquired in a 3-year interval (Figure 7). The plot shows that high levels of collaboration capacity are located between 0.1–0.6. The fact that the assortativity coefficient values were below 0.1 between 2013 and 2018 for data submission network (Table S3) can be interpreted as a below-optimal state of collaboration capacity, which coincides with the shrinking workforce and stagnant funding for young scientists in biomedical basic research (Levitt & Levitt, 2017; Pickett et al., 2015). This evidence suggests that networks that are highly assortative or disassortative would not cultivate collaboration capacity as effectively as those with moderate assortative mixing of the nodes. This leads us to speculate that researchers are more likely to attract new collaborators when they also tend to work in a moderately diverse setting. Like-wise, working with new collaborators implies a more dynamic network with stronger S&T human capital (Bozeman et al., 2001). Thus, the scatterplot in Figure 7 implies that in these networks there is an optimal state of assortative mixing.

**Figure 8.**
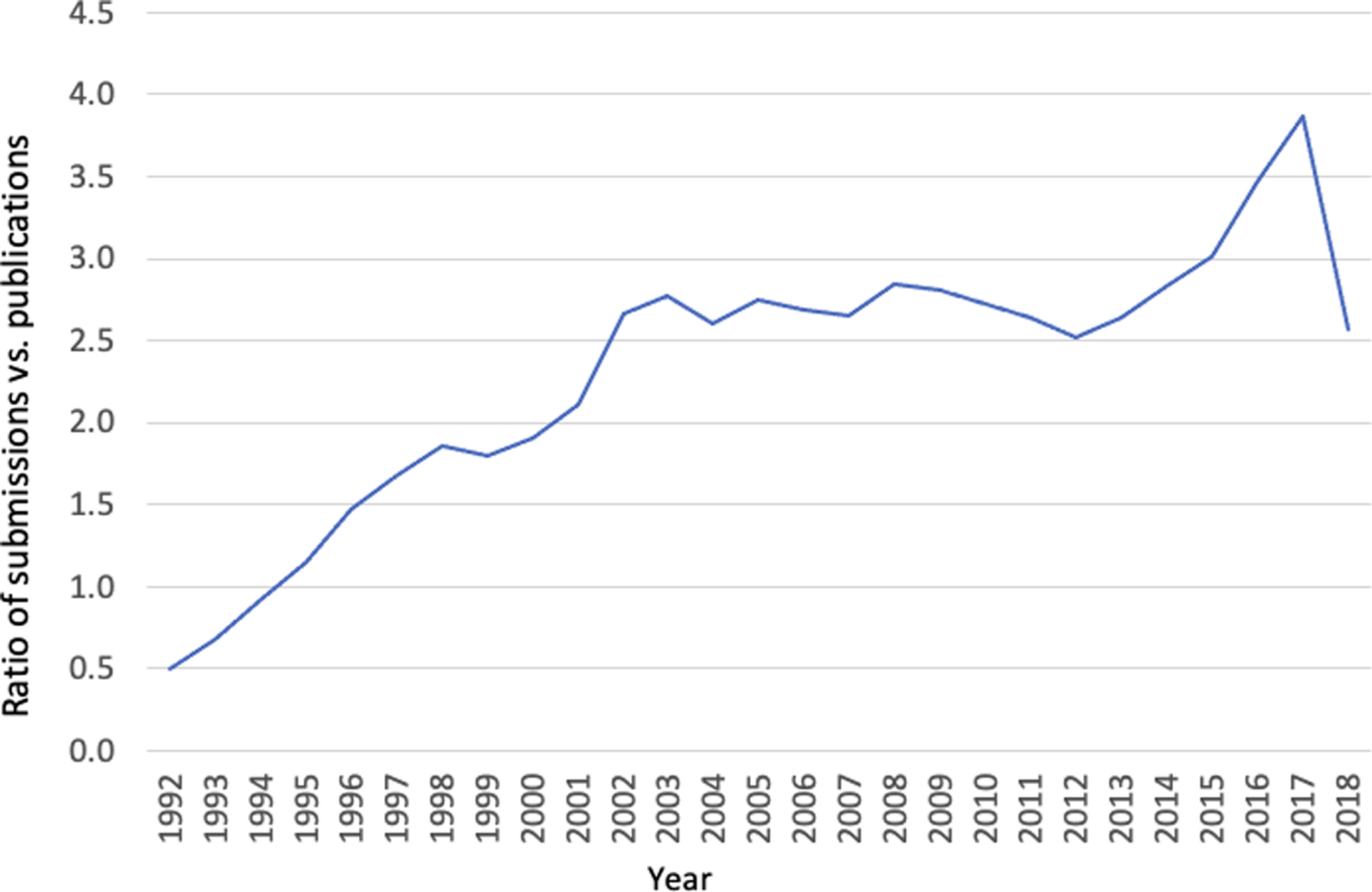
Average ratio of data submissions to publications: 1992–2018. The increment up to 2003 coincided with the Human Genome Project ending in 2003. See Table S4 for the data used to generate this plot.

The scale-free nature and heterogeneous assortative mixing in GenBank networks raises a question about how complex networks in data-intensive science can be better characterized and measured. We observed that in GenBank annotation records, a publication is often referenced in multiple data submissions. This means that, regardless of the number of base pairs involved, the number of data submissions for a publication may be an indication of the degree of data-intensiveness of the research reported in the publication. Using the ratio of the number of data submissions vs. the number of publications as a measure of data-intensiveness, the result shows a clear trend: The ratio increased steadily from 1992 through 2003 before leveling off in the next decade, which coincided with the Human Genome Project ending in 2003. Although there was a spike during 2015–16, the sudden drop in the ratio between 2017 and 2018 remains to be explored (Figure 8).

**Figure 9.**
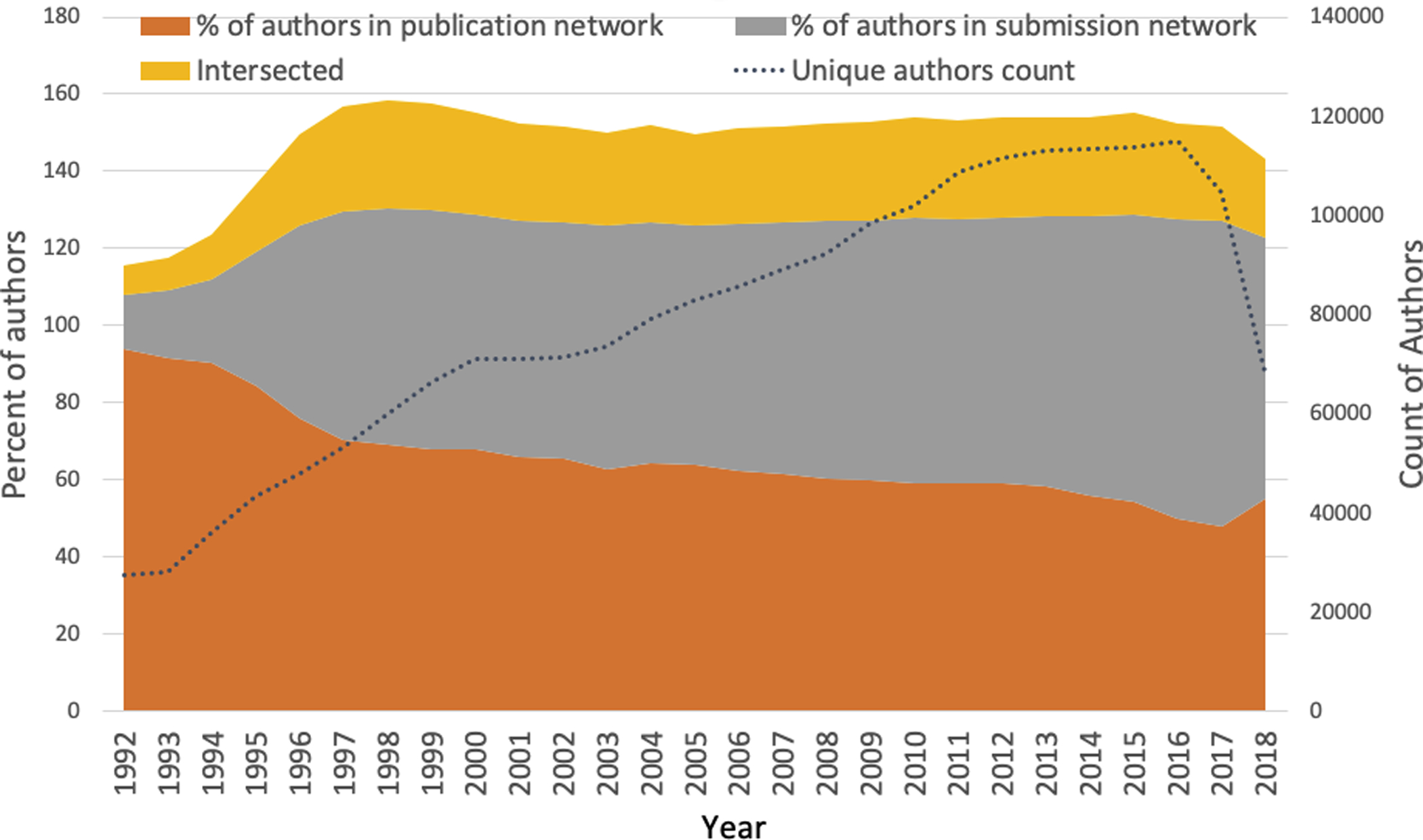
Change in the number and percentage of authors in data submission and publication networks from 1992–2018. Note that the percentage for each group does not add up to 100% because of the overlap of authors in the data submission and publication networks. The unique publication author count and unique submission author count are calculated as the total. The overlap, then, is an intersection of the two networks (publication and submission), so the “percentage intersected” includes authors from each network’s unique author counts. The data used to draw this plot are available in Table S5.

The fact that an increase in the number of data submissions (as well as authors) is involved in producing the same number of publications offers some insights into the GenBank collaboration networks and collaboration behavior in general. On the one hand, the increase in the submission-publication ratio implies that a publication required more data to support or make conclusive findings over time. This may also reflect that science has been looking at increasingly big, complex problems, hence requiring more team members for data production in support of the publications. On the other hand, the ability of a scientist to secure sufficient and highly skilled S&T human capital will significantly affect their collaboration capacity, which in turn will affect their own productivity and influence in the collaboration network. The interaction of these two factors can be observed in Figure 9, in which the percentage of authors who participated in data production (grey area) increased but those who *only* participated in publications decreased. As a percentage, authors who appeared in both data submission and publication networks remained stable after 1996.

## 6. DISCUSSION

The GenBank metadata as a new data source creates an opportunity for developing and verifying some new metrics for characterizing and measuring research collaboration networks. We used the term “collaboration capacity” (Qin, Hemsley, & Bratt, 2018) to frame the enablers of collaboration capacity—S&T human capital (Bozeman & Corley, 2004), cyberinfrastructure, and science policy—in examining collaboration networks from data production to knowledge diffusion. We assume that the greater the S&T human capital a researcher can accumulate or assemble, the more opportunity and resources they can garner to collaborate with other researchers and the more likely the S&T human capital will be used more effectively. In this sense, collaboration capacity is a framework of metrics developed for assessing the effectiveness of collaboration enablers in facilitating successful collaborations, fostering the growth of S&T human capital, and more importantly, accelerating innovations and new discoveries. Collaboration capacity is impacted by three enablers—cyberinfrastructure, S&T human capital, and science policy—and the evaluation of their impacts requires a set of metrics that can operationalize the key aspects that can reflect the impact of enablers.

The longitudinal GenBank metadata for data submissions and associated publications generated some new insights into research collaboration networks. The structural shift and patterns of assortative mixing in the GenBank collaboration networks exhibit the evolution history and trends of a large research community. The sharp drop in the total number of authors in the data submissions network from 2016 to 2018 resonates with the steady drop in grant-eligible young principal investigators (PIs) in NIH R01 grant awards, which has been warned to have a negative impact on national competitiveness in biomedical research (Levitt & Levitt, 2017). To verify whether any impact was generated on the data submissions, we used the data on sequences submitted to GenBank (NCBI, n.d.) to calculate the rate of increase. The results in Table S6 show that the number of sequences in yearly releases started to drop sharply in 2013 and the rate of increase has dropped to a historically low point (1.92, compared to 28.11 in 2005) in 2019. The drop in the number of sequences submitted to GenBank during 2013–2019 seems to correlate with the sharp drop in the data submission authors in our data. Although the COVID-19 pandemic reversed this trend (Table S6 shows that the numbers of sequences released in the first year of the pandemic [i.e., 2020] had skyrocketed by 83% of the previous year’s release total and the growth trend continued in 2021), the rise and fall of sequence submissions is nonetheless worthy of further investigation. Nucleotide sequence data as one of the pillars of biomedical research play a critical role in diagnosis, treatment development, and many other theoretical and clinical research areas, and the rapid development of COVID-19 vaccines is a great example.

Using metadata from data repositories to study complex collaboration networks represents a new research field, which can be labeled as “big metadata analytics” (Bratt, Hemsley et al., 2017). The significance of big metadata analytics lies in that the data sources cover a much larger part of a research life cycle from data to publications and can even be expanded to patents. These metadata are traces left from different stages of a research life cycle and can be valuable sources for not only enhancing research reproducibility but also uncovering characteristics and patterns of collaboration networks to help us understand better the effectiveness of collaboration capacity enablers and their impact on the transition from data to knowledge. Currently, we have integrated NIH funding data for the same period as well as matched patent metadata from the U.S. Patent and Trademark Office with those in the GenBank metadata collection to conduct further examination on questions arising from the analysis already conducted. For example, we identified nodes that had consistently high performance, from low to high performance, from high to low performance, and consistently low performance based on the number of publications and data submissions. We observed that an author with performance from low to high involved a transition from a major involvement in data submissions or equal share in data submission and publication to a major involvement in publication and less involvement in data submission. These findings raise more questions for further analysis on questions such the following: “What are some of the characteristics of these categories of different performance levels?” and “How was collaboration capacity and funding associated with the level of performance?.” The answers to questions of this nature will provide new understandings of the complex collaboration networks in data-intensive science.

## 7. CONCLUSION

The longitudinal GenBank metadata presents the evolution history of complex collaboration networks that have the properties of scale-free and power law distribution. The decrease in clustering coefficient indicated a shift from a primarily hierarchical structure to a flatter structure in GenBank collaboration networks. The analysis shows that there was an optimal assortative mixing score range for collaboration capacity. The empirical evidence in flattening network structures, increasing data collaborations, and diverse assortative mixing in this large, global-scale research community makes big metadata analytics a promising research field for exploring a fuller picture of research collaboration and science research enterprise that attests the effectiveness of utilizing S&T human capital and cyberinfrastructure as well as the impact of science policy.

## Supplementary Material

GenBank Network Degree Distribution 1992-2018

Structural Shift in Collaboration Networks: 1992-2018

Supplementary Materials for The Structural Shift and Collaboration Capacity in GenBank Networks: A Longitudinal Study

## Figures and Tables

**Table 1.** Summary of observations on the submission authors, publication authors, and the principal investigators in infectious disease related GenBank records

Year	Number of observations	Category 1: Submission author in publication		Category 2: PI in publication		Category 3: PI in submission		Yes for all three categories
		No	Yes	No	Yes	No	Yes	
1997	19	3	16	8	11	11	8	8
2006	19		19	8	11	8	11	11
2012–2014	17	1	16	2	15	9	8	0

## Data Availability

See the Supplementary Materials.
