# Supplementary material for: The structural shift and collaboration capacity in GenBank Networks: A longitudinal study
Source: Quant Sci Stud. Author manuscript; Available in PMC 2022 Apr 15. (PMC9012484; doi:10.1162/qss_a_00181)
Supplement: Supplementary Materials for The Structural Shift and Collaboration Capacity in GenBank Networks: A Longitudinal Study [file NIHMS1795165-supplement-Supplementary_Materials_for_The_Structural_Shift_and_Collaboration_Capacity_in_GenBank_Networks__A_Longitudinal_Study.pdf]

Supplementary Materials for

The Structural Shift and Collaboration Capacity in GenBank Networks: A Longitudinal Study

Jian Qin[1]*, Jeff Hemsley[1], Sarah E. Bratt[1]

[1] School of Information Studies, Syracuse University

*: Correspondence to: jqin@syr.edu

This file includes:

Figure S1
Tables S1 to S6
Captions for Movies S1 to S2

Other Supplementary Materials for this manuscript include the following:

Movie S1: Degree distribution from 1992-2018:
https://doi.org/10.7910/DVN/TMGAYB

Movie S2: Structural shift in GenBank collaboration networks: 1992-2018:
https://doi.org/10.7910/DVN/O078UE

External dataset S1: Data files used to generate Figure S1 and Figure 3:
- data submission network graph: https://doi.org/10.7910/DVN/4QUAXY
- publication network graph: https://doi.org/10.7910/DVN/YGWKLA

External dataset 2: Data files used to generate Figure 5:
https://doi.org/10.7910/DVN/ZRVK1L

External dataset 3: Data files used to generate Figure 6:
https://doi.org/10.7910/DVN/JDHB6V

Figure S1. Distribution change in GenBank submission networks: 1992-2018. Degree centrality plotted on the y-axis with network nodes sorted from largest to smallest on the x-axis. Point colors reflect the networks nodes are in. (Red: data submission; blue: publication; purple: both) The highest degree nodes in 2017 and 2018 (red) may reflect a professionalization of data submission administration. A larger size of these yearly degree distribution changes can be seen from Movie S1.

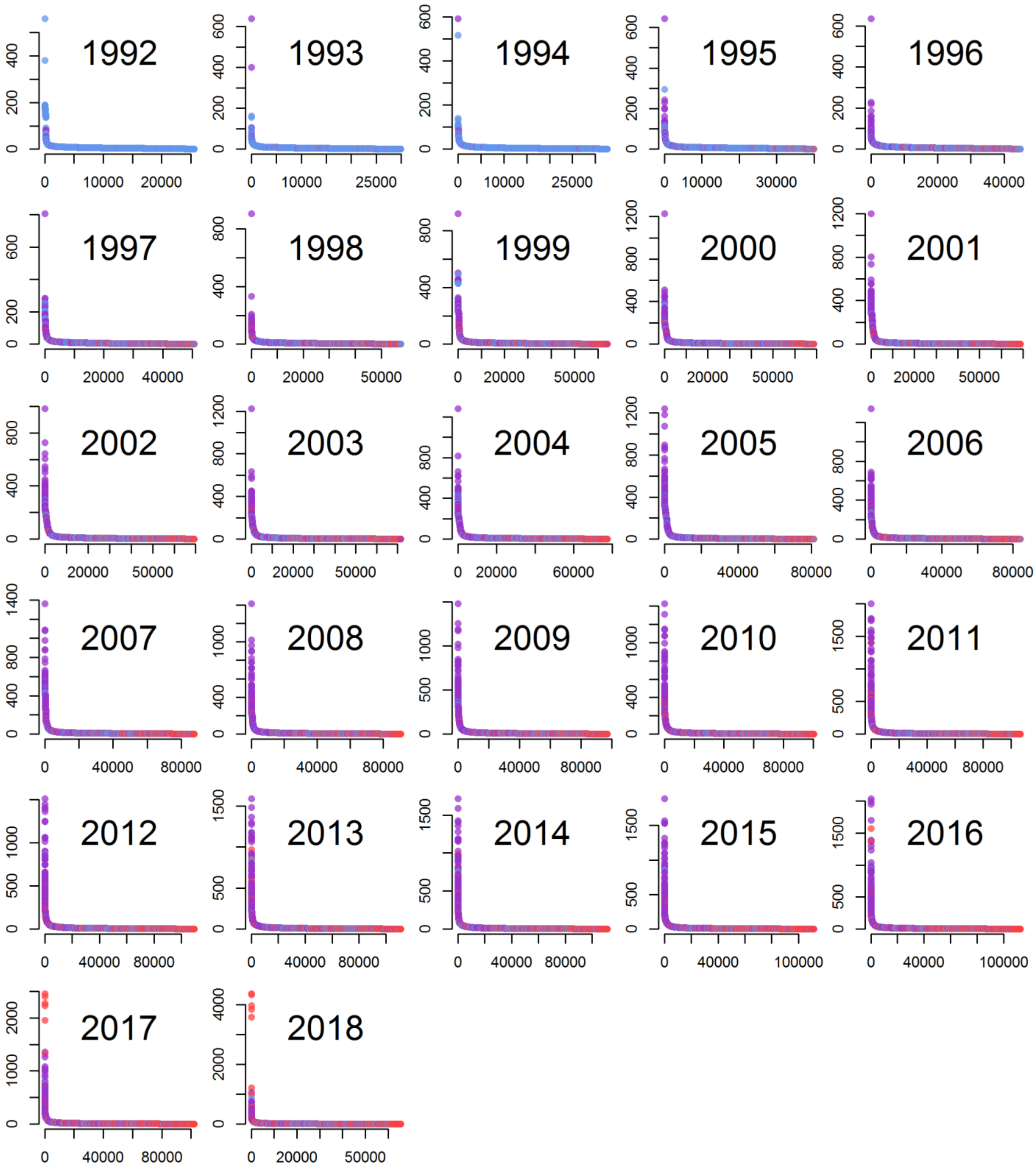
1992
1993
1994
1995
1996
1997
1998
1999
2000
2001
2002
2003
2004
2005
2006
2007
2008
2009
2010
2011
2012
2013
2014
2015
2016
2017
2018

Table S1. Power law key property (alpha and p value for Kolmogorov-Smirnov (KS) test) and mean degree distributions for submission and publication networks from 1992-2018

Year	Publication networks			Data submission networks		
	alpha	KS.p	Mean degree	alpha	KS.p	Mean degree
1992	2.966	0.000	3.38	16.893	1.000	2.26
1993	3.335	0.109	2.97	7.983	0.455	2.17
1994	3.012	0.110	3.25	3.568	0.251	2.42
1995	3.097	0.003	3.34	3.096	0.002	2.25
1996	3.076	0.052	3.43	3.169	0.222	2.37
1997	2.777	0.003	4.01	2.940	0.001	2.73
1998	3.325	0.000	3.56	2.808	0.001	2.74
1999	2.560	0.000	4.86	2.754	0.001	2.92
2000	2.568	0.000	4.96	2.458	0.000	3.41
2001	2.403	0.000	5.86	2.294	0.000	3.98
2002	2.456	0.000	5.22	2.372	0.000	4.13
2003	2.452	0.000	5.18	2.388	0.000	4.06
2004	2.383	0.000	5.8	2.455	0.000	4.07
2005	2.269	0.000	7.47	2.544	0.000	3.77
2006	2.483	0.000	5.53	2.571	0.000	3.80
2007	2.394	0.000	6.68	2.454	0.000	4.04
2008	2.578	0.206	5.29	2.527	0.000	4.26
2009	2.450	0.000	6.26	2.452	0.000	4.37
2010	2.524	0.000	5.66	2.428	0.000	4.50
2011	2.378	0.000	6.44	2.317	0.000	5.24
2012	2.514	0.007	5.87	2.427	0.033	4.79
2013	2.527	0.041	6.11	2.435	0.000	4.74
2014	2.533	0.039	5.95	2.504	0.009	4.61
2015	2.488	0.018	6.27	2.469	0.014	4.72
2016	2.501	0.002	6.34	2.491	0.002	4.80
2017	2.443	0.030	6.51	2.542	0.000	4.84
2018	2.697	0.000	5.59	2.240	0.000	5.70

Table S2. Overall properties of GenBank networks

Year	All nodes	All edges	Nodes in Giant component	% nodes in giant component	edges in giant component	% edges in giant component	Alpha for giant component	K-S goodness-of-fit test	*p* value
1992	25531	86292	11163	43.723	56243	65.178	2.750	0.045	0.000
1993	29849	88716	13976	46.822	55120	62.131	3.201	0.024	0.130
1994	32531	106033	17033	52.359	73586	69.399	2.885	0.017	0.290
1995	39890	131653	22442	56.260	95657	72.658	2.910	0.019	0.100
1996	44788	148494	27155	60.630	113923	76.719	2.929	0.025	0.000
1997	50585	195058	32295	63.843	159555	81.799	2.642	0.016	0.009
1998	57443	204778	37985	66.126	167331	81.713	3.206	0.022	0.288
1999	63895	289652	44292	69.320	251971	86.991	2.510	0.023	0.000
2000	68653	326752	48170	70.164	287467	87.977	2.387	0.023	0.000
2001	68683	389224	48435	70.520	349706	89.847	2.241	0.027	0.000
2002	69193	368510	48565	70.188	327008	88.738	2.303	0.027	0.000
2003	71440	368506	51187	71.650	328058	89.024	2.299	0.017	0.000
2004	77377	435426	57317	74.075	393655	90.407	2.274	0.020	0.000
2005	81236	528517	61249	75.396	486154	91.985	2.195	0.028	0.000
2006	84018	446084	63756	75.884	402870	90.313	2.370	0.012	0.000
2007	87441	534114	68017	77.786	493397	92.377	2.310	0.015	0.000
2008	90639	487595	71851	79.272	449305	92.147	2.428	0.010	0.005
2009	96831	582240	77556	80.094	541608	93.021	2.345	0.012	0.000
2010	100165	571200	80768	80.635	530495	92.874	2.400	0.016	0.000
2011	106517	701572	87622	82.261	662437	94.422	2.256	0.013	0.000
2012	109244	643984	89756	82.161	602863	93.615	2.408	0.008	0.042
2013	110824	670128	90786	81.919	628183	93.741	2.370	0.014	0.000
2014	111034	646130	89207	80.342	597747	92.512	2.448	0.016	0.000
2015	111211	668485	88903	79.941	619917	92.735	2.391	0.011	0.002
2016	112472	673691	89371	79.461	625166	92.797	2.436	0.011	0.004
2017	101995	607351	77906	76.382	555653	91.488	2.480	0.016	0.000
2018	65463	401760	44034	67.265	349008	86.870	2.534	0.022	0.000

Table S3. Clustering and assortativity coefficients for data submission and publication networks, 1992-2018. The yearly coefficient values are the average for all nodes in each network. These are the data used to draw Figure S4.

**year**	Clustering coefficient for data submission network	Assortativity for data submission network	Clustering coefficient for publication network	Assortativity for publication network
1992	0.823	0.150	0.813	0.649
1993	0.551	-0.265	0.452	0.012
1994	0.703	-0.098	0.600	0.144
1995	0.784	0.030	0.583	0.205
1996	0.672	0.152	0.536	0.044
1997	0.731	0.461	0.725	0.598
1998	0.727	0.639	0.489	0.020
1999	0.665	0.555	0.835	0.808
2000	0.723	0.543	0.737	0.607
2001	0.838	0.725	0.869	0.836
2002	0.810	0.761	0.722	0.634
2003	0.727	0.640	0.767	0.593
2004	0.733	0.653	0.758	0.680
2005	0.716	0.620	0.791	0.656
2006	0.504	0.210	0.720	0.630
2007	0.523	0.247	0.871	0.867
2008	0.572	0.387	0.596	0.415
2009	0.433	0.202	0.723	0.599
2010	0.457	0.265	0.486	0.234
2011	0.288	0.005	0.523	0.218
2012	0.416	0.172	0.352	0.128
2013	0.303	0.046	0.391	0.170
2014	0.292	0.044	0.296	0.119
2015	0.304	0.031	0.406	0.141
2016	0.297	0.014	0.447	0.179
2017	0.263	-0.013	0.473	0.181
2018	0.150	-0.066	0.386	0.118

Table S4. Ratio of data submissions vs. publications

Year	Number of data submissions	Number of publications	Ratio of submissions vs. publications
1992	4407	8792	0.501
1993	6778	9980	0.679
1994	9882	10694	0.924
1995	13323	11613	1.147
1996	16581	11245	1.475
1997	19610	11688	1.678
1998	24379	13111	1.859
1999	27967	15573	1.796
2000	31521	16584	1.901
2001	32388	15324	2.114
2002	40754	15290	2.665
2003	40099	14449	2.775
2004	40925	15706	2.606
2005	43915	15959	2.752
2006	44747	16675	2.683
2007	46406	17457	2.658
2008	49365	17331	2.848
2009	51473	18307	2.812
2010	50383	18511	2.722
2011	50658	19180	2.641
2012	54145	21478	2.521
2013	55281	20923	2.642
2014	57717	20377	2.832
2015	57832	19186	3.014
2016	59073	17019	3.471
2017	54818	14175	3.867
2018	26002	10117	2.570

Table S5. Overlapping authors in GenBank networks. Data used to generate Figure 8

Year	Number of unique authors in publication network	Number of unique authors in data submission network	Number of intersected authors	Total number of unique authors	% of authors in publication network	% of authors in data submission network	% of intersected authors
1992	25399	437	305	25836	98.31	1.69	1.18
1993	29101	1457	709	30558	95.23	4.77	2.32
1994	32212	826	507	33038	97.50	2.50	1.53
1995	35637	7925	3672	43562	81.81	18.19	8.43
1996	35244	17686	8142	52930	66.59	33.41	15.38
1997	36317	25923	11655	62240	58.35	41.65	18.73
1998	40064	31370	13991	71434	56.09	43.91	19.59
1999	43364	35898	15367	79262	54.71	45.29	19.39
2000	46710	37874	15931	84584	55.22	44.78	18.83
2001	45285	38657	15259	83942	53.95	46.05	18.18
2002	45195	39320	15322	84515	53.48	46.52	18.13
2003	44638	42218	15416	86856	51.39	48.61	17.75
2004	49570	45275	17468	94845	52.26	47.74	18.42
2005	51494	47423	17681	98917	52.06	47.94	17.87
2006	51764	50988	18734	102752	50.38	49.62	18.23
2007	53420	53889	19868	107309	49.78	50.22	18.51
2008	54046	57490	20897	111536	48.46	51.54	18.74
2009	57546	61924	22639	119470	48.17	51.83	18.95
2010	58694	65626	24155	124320	47.21	52.79	19.43
2011	62269	69364	25116	131633	47.31	52.69	19.08
2012	64088	71296	26140	135384	47.34	52.66	19.31
2013	63714	73408	26298	137122	46.47	53.53	19.18
2014	61191	76174	26331	137365	44.55	55.45	19.17
2015	59639	78576	27004	138215	43.15	56.85	19.54
2016	55165	83053	25746	138218	39.91	60.09	18.63
2017	47953	76934	22892	124887	38.40	61.60	18.33
2018	35848	41855	12240	77703	46.13	53.87	15.75

Table S6. Sequences submitted to GenBank and rate of increase since 2013

Year	Sequences	Increment to previous year	Rate of increase
2004	40,604,319		
2005	52,016,762	11,412,443	28.11
2006	64,893,747	12,876,985	24.76
2007	80,388,382	15,494,635	23.88
2008	98,868,465	18,480,083	22.99
2009	112,910,950	14,042,485	14.2
2010	129,902,276	16,991,326	15.05
2011	146,413,798	16,511,522	12.71
2012	161,140,325	14,726,527	10.06
2013	169,331,407	8,191,082	5.08
2014	179,295,769	9,964,362	5.88
2015	189,232,925	9,937,156	5.54
2016	198,565,475	9,332,550	4.93
2017	206,293,625	7,728,150	3.89
2018	211,281,415	4,987,790	2.42
2019	215,333,020	4,051,605	1.92
2020	1,309,033,549	1,093,700,529	83.55
2021	1,381,436,348	72,402,799	5.24

Source: Data in this table come from GenBank and WGS Statistics: https://www.ncbi.nlm.nih.gov/genbank/statistics/.

Movie S1.

Distribution change in GenBank submission networks: 1992-2018. Degree centrality plotted on the y-axis with network nodes sorted from largest to smallest on the x-axis. Point colors reflect the networks nodes are in. (Red: data submission; blue: publication; purple: both) The highest degree nodes in 2017 and 2018 (red) may reflect a professionalization of data submission administration. The visualizations were generated with External dataset S1: data submission network graph: https://doi.org/10.7910/DVN/4QUAXY and publication network graph: https://doi.org/10.7910/DVN/YGWKLA.

Movie S2.

GenBank network visualization from 1992-2018: Each network represents one year of the data and includes the merged data submission and publication co-author networks. Nodes that only showed up in the publication network are blue with green links. Nodes that only showed up in the data submission network are dark red, with red links. Nodes that showed up in both networks are purple with dark purple links between them. To observe the main structures, we are focused on the giant component for each year, thus isolates and disconnected clusters have been

removed. The visualizations were generated with external dataset S1: data submission network graph: https://doi.org/10.7910/DVN/4QUAXY and publication network graph: https://doi.org/10.7910/DVN/YGWKLA.
